# Supplementary material for: MetaQTL: a package of new computational methods for the meta-analysis of QTL mapping experiments
Source: BMC Bioinformatics. 2007 Feb 8;8:49. doi: 10.1186/1471-2105-8-49 (PMC1808479; doi:10.1186/1471-2105-8-49)
Supplement: Additional File 7 — MetaQTL Package : jar file and tutorial. This Zip archive contains both the MetaQTL JAR file and the files of the tutorial. [file 1471-2105-8-49-S7.zip › org.inra.metaqtl/doc/org/metaqtl/MetaMap.html]

MetaMap


|  |  |  |
| --- | --- | --- |
| **Overview** \| **Package** \| **Class** \| **Use** \| **Tree** \| **Deprecated** \| **Index** \| **Help** | |  |
| **PREV CLASS**   **NEXT CLASS** | **FRAMES**    **NO FRAMES**     **All Classes** |  |
| SUMMARY: NESTED \| FIELD \| CONSTR \| METHOD | DETAIL: FIELD \| CONSTR \| METHOD |  |


---


## org.metaqtl Class MetaMap

```
java.lang.Object
  org.metaqtl.MetaMap
```

---

``` public class MetaMap extends java.lang.Object ```

A `MetaMap` is a bundle of `MetaChrom` objects.

---

| **Field Summary** | |
| --- | --- |
| `MetaChrom[]` | `chromosomes`             The meta chromosomes. |
| `int` | `nchr`             The number of meta chromosmes. |


**Constructor Summary**

`MetaMap(int nchr)`
  
          Creates a new instance of `MetaMap`
which will contain `nchr` `MetaChrom`
objects.


**Method Summary**

**Methods inherited from class java.lang.Object**

`clone, equals, finalize, getClass, hashCode, notify, notifyAll, toString, wait, wait, wait`

**Field Detail**

### nchr

```
public int nchr
```

:   The number of meta chromosmes.

---


### chromosomes

```
public MetaChrom[] chromosomes
```

:   The meta chromosomes.


**Constructor Detail**

### MetaMap

```
public MetaMap(int nchr)
```

:   Creates a new instance of `MetaMap`
    which will contain `nchr` `MetaChrom`
    objects.

    **Parameters:**: `nchr` - the number of meta chromsomes.


---


|  |  |  |
| --- | --- | --- |
| **Overview** \| **Package** \| **Class** \| **Use** \| **Tree** \| **Deprecated** \| **Index** \| **Help** | |  |
| **PREV CLASS**   **NEXT CLASS** | **FRAMES**    **NO FRAMES**     **All Classes** |  |
| SUMMARY: NESTED \| FIELD \| CONSTR \| METHOD | DETAIL: FIELD \| CONSTR \| METHOD |  |


---
